# Net costs of breast cancer in Colombia: a cost-of-illness study based on administrative claims databases

**DOI:** 10.1186/s12962-024-00562-z

**Published:** 2024-07-02

**Authors:** Gabriel Fernando Torres, Brigitte Alejandra Alarcón, Juan Manuel Reyes-Sanchez, Natalia Castaño-Gamboa, Giancarlo Buitrago

**Affiliations:** 1 Instituto de Investigaciones Clínicas, Universidad Nacional de Colombia, Carrera 45 # 26-85, Bogotá, 111321 Colombia; 2 Pfizer S.A.S, Av. Suba #95-66, Bogotá, 112111 Colombia; 3 Hospital Universitario Nacional, Calle 44 # 59-75, Bogotá, 111321 Colombia

**Keywords:** Breast cancer, Cost-of-illness study, Net costs

## Abstract

**Background:**

Breast Cancer (BC) is associated with substantial costs of healthcare; however, real-world data regarding these costs in Colombia is scarce. The contributory regime provides healthcare services to formal workers and their dependents and covers almost half of the population in Colombia. This study aims to describe the net costs of healthcare in women with BC covered by the contributory regime in Colombia in 2019 from the perspective of the Colombian Health System.

**Methods:**

The main data source was the Capitation Sufficiency Database, an administrative database that contains patient-level data on consumption of services included in the National Formulary (PBS, in Spanish Plan de Beneficios en Salud). Data on consumption of services not included in the PBS (non-PBS) were calculated using aggregated data from MIPRES database. All direct costs incurred by prevalent cases of BC, from January 1 to December 31, 2019, were included in the analysis. The net costs of the disease were estimated by multiplying the marginal cost and the expected number of cases with BC by region and age group. Marginal costs were defined as the costs of services delivered to patients with BC after subtracting the expected costs of health services due to age, comorbidity burden or region of residence. To calculate these costs, we used Propensity Score Matching in the main analysis. All costs were expressed in 2019 international dollars. Productivity losses, transportation expenses, and caregiving costs were not included.

**Results:**

A total of 46,148 patients with BC were identified. Total net costs were $387 million (95% CI $377 to $396 million), 60% associated with non-PBS services. Marginal costs were $8,366 (95% Confidence Interval $8,170 to $8,573), with substantial variations between regions age groups (from $3,919 for older patients in the Amazonia region to $10,070 for younger patients in the Pacific region). The costs for PBS services were higher for ambulatory services and for patients who died during 2020.

**Conclusions:**

BC imposes a substantial economic burden for the Colombian Health System with important variations in net costs between regions and age groups. Patients near death and ambulatory services were associated with higher costs of healthcare.

**Supplementary Information:**

The online version contains supplementary material available at 10.1186/s12962-024-00562-z.

## Introduction

Breast cancer (BC) is the most common malignancy and one of the leading causes of death among women worldwide [1]. According to information published by the Ministry of Health, 6,593 incident cases of BC were diagnosed during 2020 in Colombia, and almost one-third of all these cases were diagnosed at stage III or IV [2]. Although incidence rates of BC in high-income countries have usually been higher, incidence rates in low- and middle-income countries (LMICs) have been rising by up to 5% per year [3]. This continuous increase in incidence adds up to the high mortality burden associated with BC in patients from LMICs, largely attributed to limited diagnostic and treatment capacity in these settings [4, 5]. In addition to the direct effects of the disease on health, BC also increases financial pressures on most health care systems [6, 7]. This greater financial burden displaces investments from other programs within health systems.

Cost-of-illness (COI) studies seek to evaluate the economic burden that diseases impose on society in terms of the use of health care resources [8]. This information can help estimate the magnitude of diseases in monetary terms, justify preventive or therapeutic strategies, and provide an economic framework for the evaluation of programs, among other useful applications [9]. BC has been the subject of several COI studies [10–16]; however, there exists great variability between the estimates from these studies in Colombia. Furthermore, there is no information regarding the net costs associated to BC after adjusting for comorbidities. A failure to adjust for comorbidities increases the risk of double counting due to the attribution of all expenditures to the main diagnosis and is related to the generation of implausible large estimates of attributable costs [17].

Since 1993, Colombia has provided mandatory health insurance to all its citizens through several regimes of affiliation based on a managed care competition system [18]. Formal workers and their dependents are affiliated with the contributory regime and comprise almost 48% of the total country’s population. Despite their regimen of affiliation, all individuals are entitled to receive all health services prescribed by authorized health professionals, included or not, in the National Health Benefit Package (HBP). In theory, the HBP contains all diagnostic, therapeutic and rehabilitation services to which all individuals are entitled to within the Health System, under a cost-effectiveness approach [18]. In practice, the Ministry of Health undertakes a yearly updating process which continuously expands the list of services included in the HBP. However, new technologies, as well as off-label uses of some included drugs are, at least temporarily, not included in the HBP. Given the continuous growth of available therapeutic options, the cost of non-HBP services frequently takes a substantial share of the total costs of care in cancer [19]. According to data published by the Ministry of Health, almost 90% of non-HBP costs within the health system are related to the delivery of non-HBP drugs [20]. Most of these services are provided by health maintenance organizations with mixed public and private ownership, and they are reimbursed by the Ministry of Health through a system of risk-adjusted capitation payments.

This study aims to estimate the net costs of BC in women affiliated with the contributory regime in Colombia in 2019. According to Barlow, net cost is defined as *“the difference between the mean costs for cancer patients and for patients without cancer who are otherwise comparable”* [21]. All costs borne by the health system, medications, inpatient and outpatient services, and all diagnostic and surgical procedures, regardless of disease stage or histological classification, are included in this study. Costs not borne by the health system, such as productivity losses, out-of-pocket expenditures, transportation costs or caregiving, are not included.

## Methods

### Setting and design

This study uses a retrospective COI approach based on prevalent cases to establish the net costs of BC borne by the contributory regime in 2019. For services included in the HBP (HBP services), net costs are estimated using individual-patient data from existing administrative databases. For services not included in the HBP (non-HBP services), quantities and prices are extracted from aggregated data using the public information system provided by the Ministry of Health (SISPRO from the Spanish *Sistema Integrado de Información de la Protección Social*) and available tariff manuals. Finally, total net costs are calculated as the sum of the costs of HBP and non-HBP services. All costs are transformed to 2019 International US dollars using the Purchasing Power Parity conversion factor for Colombia published by The World Bank (1,343.6 Colombian pesos per international dollar) [22]. All costs are expressed as the mean monetary values paid by the health system for the provision of health services to patients with BC during 2019, regardless of the time since diagnosis or the severity of the disease. Given previous reports of substantial differences between costs estimates for patients with recent diagnosis or in proximity to death, this study explores 2019 mean costs for patients in the study cohort who received a recent diagnosis (defined as those with a first diagnosis of BC during 2018), and for patients with proximity to death (defined as those who died during the first semester of 2020). All analyses are made using Stata MP^®^ 14.0 and Microsoft^®^ Excel^®^ for Microsoft 365 MSO licensed to the National University of Colombia.

### Data sources

This study uses data from existing administrative databases within the health system. The main source of data is the Database for the Study of the Sufficiency of the Capitation Unit (UPC from the Spanish *Base de datos para el estudio de la Unidad por Capitación*). The UPC database is used by the Ministry of Health to adjust the capitation payments received by HMOs according to the level of use of HBP services of their affiliates. The available UPC database reports patient-level data on the use of HBP services from approximately 80% of the contributory regime and includes the costs paid by the health system, the diagnosis associated with each service using the International Classification of Diseases (ICD-10), and the 10th revision jointly with the basic demographic characteristics. The UPC database is reported by HMOs to the Ministry of Health, and its data are collected at the point of delivery by health care providers. The process of data cleaning and validation of the UPC database is described in detail by Bolivar et al. [23].

All individuals in the UPC database are identified using an anonymized identification number that allows us to link their data to the Unique Registry of Affiliates (RUAF from the Spanish *Registro Único de Afiliados*) and the Unique Database of Affiliation (BDUA from the Spanish *Base de Datos Única de Afiliación*). RUAF allows health professionals to report data on all births and deaths, and its data are key to estimating, among others, mortality indicators of interest for the Colombian Government. The BDUA contains data from all individuals affiliated with the health system, and its data are essential for all reimbursements made by the Ministry of Health to HMOs [24]. Finally, this study uses aggregated data from the My Prescription Database (MIPRES from the Spanish *Mi Prescripción*) available through SISPRO [25]. MIPRES is a web tool that allows health professionals to report the prescription of non-PBS services, and it supports all claims for non-PBS services made by the HMOs.

### Identification of patients

To identify patients with BC affiliated with the contributory regime, this study used the electronic algorithm previously validated by Saldaña et al. [26]

Saldana et al. compared the incidence estimates of breast, stomach, and colorectal cancer obtained by several electronic algorithms in the UPC database with the estimates published by a prospective, multicentric, cancer registry in Colombia known as Infocancer. The electronic algorithms that yielded the incidence estimates nearest to the ones published by Infocancer were selected as the recommended algorithms for research within the UPC database.

For BC, the persistence of ICD-10 codes for at least four months and at least one BC-specific procedure (the “specific” algorithm) was selected as the recommended electronic algorithm and was used in this study for the main analysis. To estimate the robustness of these results to changes in the electronic algorithm, we calculated the prevalence estimates and the number of cases detected using the persistence of ICD-10 codes for at least four months without the criteria for BC-specific procedures (the “sensitive” algorithm). The full electronic algorithms used in this study are described in Table S1 in the additional file.

All women who fulfilled the specific algorithm at any time from 2015 to 2019 and received at least one health care service during 2019, regardless of the stage of the disease or its histological classification, were included in the main cohort and classified as “exposed”. Unexposed individuals were selected among all individuals affiliated with the contributory regime who did not receive any ICD-10 code of BC from 2015 to 2019 and received at least one health care service for any other reason during 2019. Data regarding unexposed individuals without any consumption of health services during 2019 (i.e., non-users), and who did not receive any ICD-10 code of BC from 2015 to 2019, were collected from the corresponding BDUA database.

Finally, prevalence estimates were calculated as follows:

$${P}_{ar}=\frac{{n}_{ar}}{{N}_{ar}}\times \text{1,000}$$

where ${P}_{ar}$ is the prevalence estimate per age group a, and region r, ${n}_{ar}$ is the number of exposed individuals (i.e. women who received at least one BC related health service per month for at least four different months at any time from 2015 to 2019), who were alive on January 1st, 2019 and identified in the UPC database; and ${N}_{ar}$ is the number of women affiliated to the EAPBs with data available in the UPC database and identified using BDUA.

All costs accrued by exposed and unexposed individuals and borne by the health system, from January 1st to December 31st, 2019, were considered in the analysis. Given that, Colombia provides compulsory insurance coverage for all its citizens and that the UPC database contains data on most individuals affiliated with the Contributory regime, no administrative censorship or losses to follow-up were considered in the cohort.

To explore plausible differences in costs during 2019 between women within the first year of diagnosis of BC or at the end of life, this study identified the subgroups of patients who received their first diagnosis of BC during 2018 or who died during the first semester of 2020 within the main cohort. Given that the available UPC database contains data regarding approximately 80% of the individuals affiliated with the contributory regime, this study assumes that the individuals in the remaining 20% of the contributory regime present a similar distribution of disease conditional prevalence, demographic characteristics, and costs.

### Estimation of net costs associated to non-HBP services

To estimate the net costs associated to the delivery of non-HBP services, this study uses data on quantities from the MIPRES database through SISPRO and data on prices from the national tariff manuals [25, 27, 28]. Equation 4 summarizes the approach used to calculate the costs of non-HBP services:

1 $$Cost\, nonHBP= \sum _{region}\sum _{age}\sum _{service}{\stackrel{-}{q}}_{iras}\times {p}_{s}\times {n}_{ra}$$

where $\mathop q\limits^ -$_*iras*_ is the mean number of non-HBP services delivered per patient by region, age group and service, *p*_*s*_ is the price of each service according to the data published by the Ministry of Health in national tariff manuals, and *n*_*ra*_ is the expected number of patients per region and age group. To decrease the risks of a misallocation of services to the main disease, this study only considers the costs associated with the delivery of targeted therapies (that includes immunotherapy, hormonal therapy, and standard chemotherapy) in this category. We believe that this is a valid assumption since most non-HBP costs are caused by the delivery of targeted therapies, and these are rarely used for other conditions unrelated to cancer. The full list of included non-HBP therapies is shown in Table S2 in the additional file.(20). All estimates are reported with their corresponding 95% confidence intervals (95% CI), assuming a uniform distribution for prices and quantities identified in the MIPRES database as the population parameters.

### Estimation of net costs associated to HBP services

A failure to adjust for comorbidities increases the risk of double counting due to a misallocation of all expenditures to the main diagnosis. To decrease this risk, this study uses an approach based on first estimating the marginal costs of delivering HBP services after adjusting for comorbidities (τ) and then multiplying them by the expected number of individuals (n) per geographical region r and age group a. Equation 1 summarizes this approach:

2 $$Costs\, HBP=\sum _{region}\sum _{age}{\tau }_{ra}\times {n}_{ra}$$

“To estimate τ, this study uses a nearest neighbor propensity score matching (PSM) within a caliper of 1 × 10 − 3% points without replacement in the main analysis.”. PSM allows us to decrease the risk of a misallocation of costs by comparing individuals with and without a given condition, but otherwise, a similar risk of use of resources [19]. The methods used to estimate attributable costs in COI studies using PSM have been described previously [29]. In summary, propensity scores per individual *i* are defined as the conditional probability of assignment to a particular treatment (*W* = 1) versus no treatment (*W* = 0) given a vector of observed covariates *x*_*i*_:

3 $$e\left({\text{x}}_{i}\right)=pr\left({W}_{i}=1|{X}_{i}={\text{x}}_{i}\right)$$

To estimate the propensity scores, this study uses logistic regression analysis with a binary variable that indicates the disease status as the dependent variable and comorbidities (using the Charlson Comorbidity Index), age, region of residence and employment status (defined as employed or unemployed during the last 12 months) as explanatory variables. Given that data regarding income during the last 12 months is available for approximately 80% of the entire cohort only, we performed a sensitivity analysis to compare our PSM estimates for the full cohort and for the subgroup with available data regarding average income (see Table S3 in the additional file). Finally, the marginal cost (τ) is defined as the mean of the expected differences across all the matched pairs. Equation 3 summarizes this approach:

4 $${\tau }_{ra}=\frac{1}{{M}_{ra}}\sum E[{c}_{ira}-{c}_{ira}^{{\prime }}\left|e\left(x\right)\right]$$

where *M*_*ra*_ is the number of matched pairs and *c*_*ira*_ and *c’*_*ira*_ are the costs of delivering health services per individual *i* in the age *a* and region *r* group, with and without BC, respectively.

The quality of the matching was assessed by calculating the remaining differences across the covariates. Standardized differences between individuals with and without BC after matching of greater than 20% were considered unacceptable (see Table S4 in the additional file).

The robustness of estimations of marginal costs were tested using multilevel ordinary least squares (OLS) mixed models, with a random intercept for department at level two and individual affiliates at level one. The fitted mixed OLS regression model is as follows:

$$\eqalign{ COST{S_{ij}} = & {\beta _0} + {\beta _1}BREAS{T_{ij}} + {\beta _2}AG{E_{ij}} + \cr & {\beta _3}IN{S_{ij}} + {\beta _4}JO{B_{ij}} + \delta {Z_{ij}} + {u_j} + { \in _{ij}} \cr}$$

where *COSTS*_*ij*_ represents the costs accrued throughout 2019 by the individual *i* in the department *j* where most health services were delivered; *BREAST*_*ij*_ represents a dummy variable that can take values of one if the individual is classified with diagnosis of breast cancer, or zero otherwise; *AGE* represents the age in years, *INS* is a nominal variable that represents the insurer; *JOB* represents a dummy variable that can take values of one if the individual *i* was employed during the last 12 months, or zero otherwise. The vector ***Z*** includes dummies for all comorbidities defined by the Charlson Comorbidity Index; *u*_*j*_ represents a random intercept that varies according to the department where most services were delivered during the study; and $\in$_*ij*_ an error term clustered by individual. The OLS model included all variables available in the dataset to describe baseline sociodemographic and clinical characteristics, therefore no variable selection strategies were used to specify the final model.

All estimates are reported with 95% confidence intervals (95% CIs) using robust standard errors and are reported by category of service and setting. Exploratory estimations of costs are made for the subgroup of patients who received the first diagnosis of BC during 2018 and for the subgroup of those who died during the first semester of 2020.

### Estimation of total net costs

Finally, total attributable costs by age group and region are calculated by summing the costs due to the delivery of HBP and non-HBP services as calculated in Eqs. 1 and 4. All estimates of total attributable costs and their corresponding 95% CI are presented by category, setting of delivery and exploratory subgroups of interest.

## Results

### Identification of patients with breast cancer in the contributory regime

**Fig. 1:**
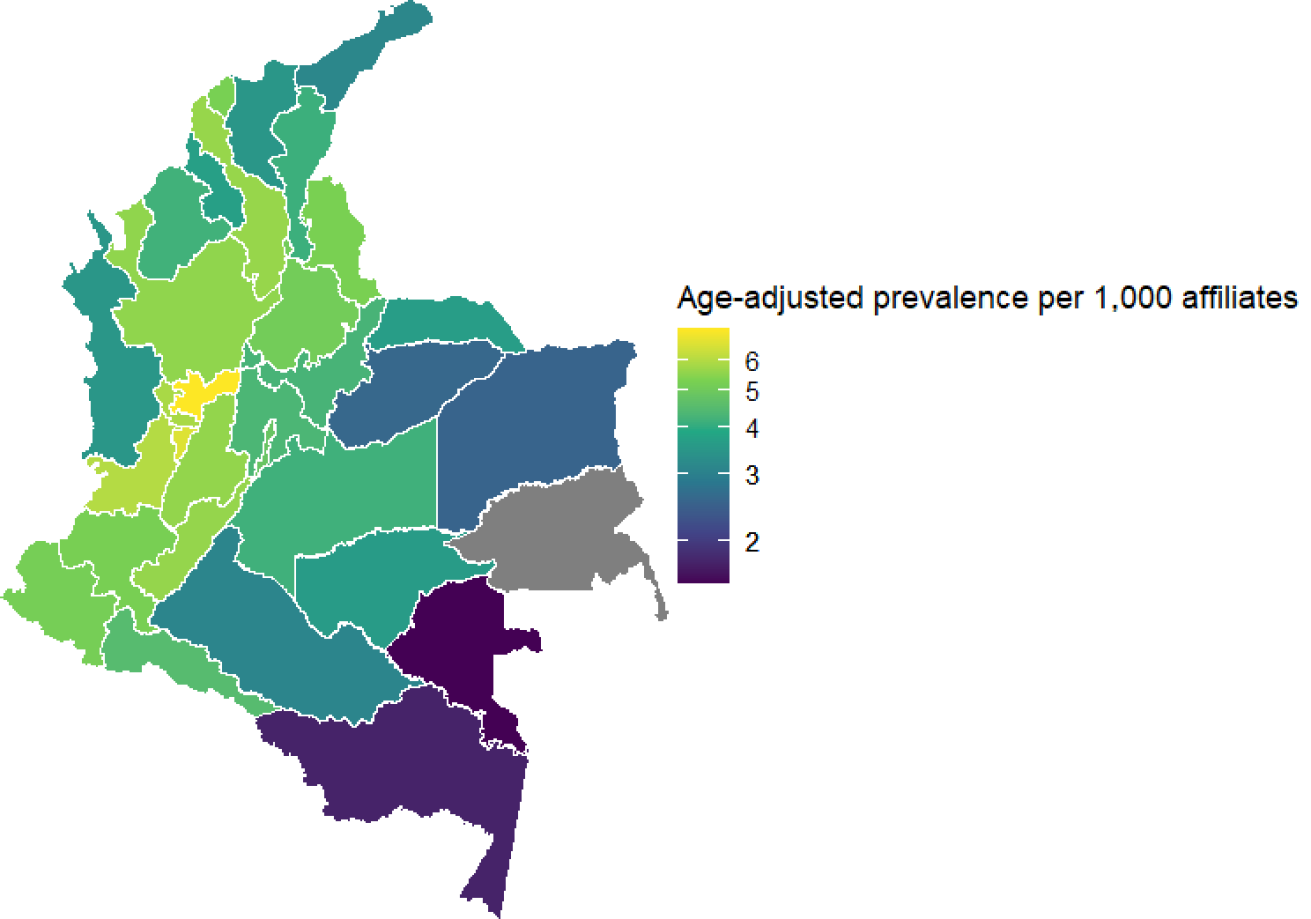
Age-adjusted prevalence of Breast Cancer in the contributory regime by department, conditional to the consumption of at least one health service during 2019

Using the specific algorithm described by Saldaña et al. [26], this study identified 40,800 women with BC in the UPC database during 2019. Assuming a similar distribution of baseline risks with women with unavailable data, 46,148 women in the contributory regime suffered from BC and used at least one health service during 2019 (see Tables S5 and S6 in the additional file). The age-adjusted prevalence conditional on the use of health services varied widely across the country, ranging from 3.8 cases per 1,000 women in Amazonia to 5.8 cases per 1,000 women in the Pacific region (see Fig. 1). The baseline characteristics of the main study cohort and the prespecified subgroups are described in Table 1. In summary, whereas most women from the main cohort were between 45 and 64 years old and had a Charlson Comorbidity Index (CCI) of I or II (including the cancer diagnosis), most women from the cohort of deceased in 2020 were 65 years old or older and had a CCI of five or more. In contrast, most women from the main cohort and all subgroups lived in Bogotá or the Central region of the country.

**Table 1:** Baseline characteristics of the main study cohort

	Main cohort (*n* = 40,800)		Diagnosis in 2018 (*n* = 5,067)		Deceased in 2020 (*n* = 833)	
	n	%	n	%	n	%
**Age (years)**						
20–44	6,469	15.86	923	18.22	107	12.85
45–64	20,804	50.99	2672	52.73	351	42.14
65 or more	13,527	33.15	1472	29.05	375	45.02
**Charlson comorbidity index**						
I – II	19,931	48.85	2943	58.08	226	27.13
III – IV	11,673	28.61	1476	29.13	257	30.85
V or more	9,196	22.54	648	12.79	350	42.02
**Presence of ICD-10 codes associated with metastatic disease**						
Yes	3,847	9.43	140	2.76	175	21.01
No	36,953	90.57	4,927	97.24	658	78.99
**Region of residence**						
Atlántica	4,606	11.29	628	12.39	114	13.69
Bogotá	11,994	29.40	1335	26.35	237	28.45
Central	12,316	30.19	1566	30.91	223	26.77
Oriental	5,216	12.78	701	13.83	114	13.69
Orinoquía	226	0.55	35	0.69	3	0.36
Pacífica	6,442	15.79	802	15.83	142	17.05

*n* number of cases

### Estimation of net costs associated to HBP services

**Fig. 2:**
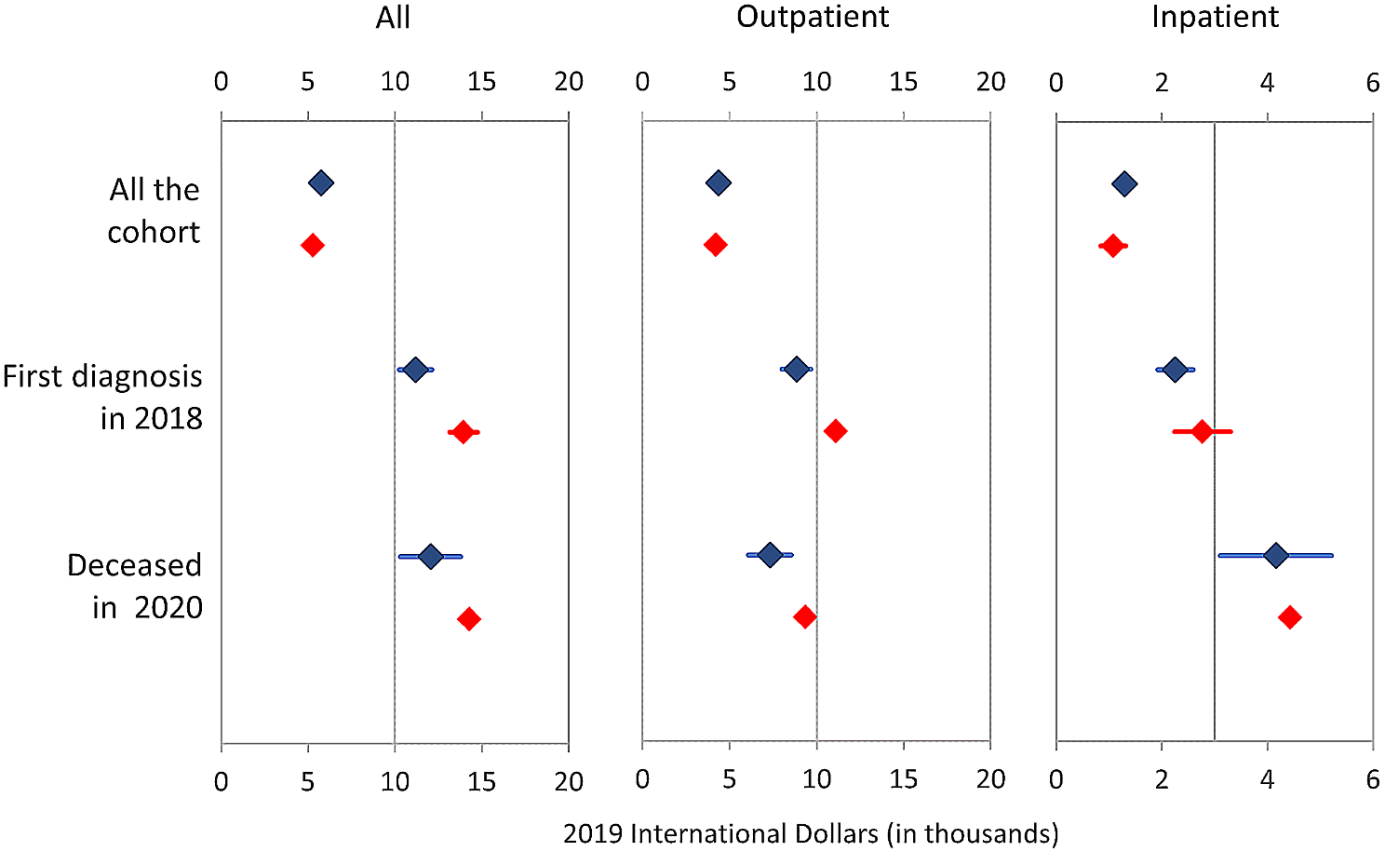
Marginal costs of delivering services included in the Health Benefit Package in women affiliated with the contributory regime in 2019 by type of model’s specification (Propensity Score Matching: blue, Ordinary Least Squares: red)

The total net costs associated with the delivery of HBP services in the contributory regime were $237.3 million (95% CI $236 to $238.8 million) during 2019. The distribution of these costs across age groups and regions is described in Table 2, and tables S5 and S6 in the additional file. In brief, the mean attributable cost of delivering HBP services in the contributory regime was $5,143 (95% CI $5,113 to $5,172). These costs were substantially different between regions, age groups and settings of delivery. Regarding regions, mean attributable costs were lower for Bogotá ($4,836, 95% CI $4,787 to $4,885) than for other regions (from $5,041 [95% CI $4,965 to $5,116] in Atlántico to $7.934 [95% CI $7,569 to $8,300] in Orinoquia). Likewise, regarding differences between age groups and settings of delivery, mean attributable costs per patient were lower for women of older ages (from $7,334 to $10,077 in the group of 20 to 44 years old to $2,756 to $4,489 in the group of 65 years old or older) and inpatient services (from $955 to $993 for inpatient services to $4,160 to $4,195 for outpatient services). The subgroups of patients with an initial diagnosis during 2018 and those who died during the first semester of 2020 showed higher mean per patient costs of care during 2019 than those in the main cohort. The results of using OLS to estimate the mean attributable costs per patient are shown in Fig. 2. No apparent differences were found between the results yielded by both model specifications. Likelihood-ratio tests comparing the two-level analysis with one-level ordinary linear regressions were highly significant for most fitted models. Additional information regarding mean attributable costs per patient by category of service is described in Table S7 and S8 in the Additional file.

**Table 2:** Mean attributable costs per patient in the Contributory Regime during 2019 by region

	*n*	Services included in the health benefit package	Services not included in the health benefit package	Total
Atlántica	5,190	5,040.62	3,280.17	8,320.95
		(4,964.94–5,116.30)	(2,904.16–3,646.38)	(7,935.88–8,705.30)
Bogotá	12,887	4,835.78	3,904.43	8,741.07
		(4,786.89–4,884.67)	(3,731.39–4,073.49)	(8,558.56–8,925.69)
Central	13,943	5,262.80	2,400.69	7,661.57
		(5,213.57–5,312.03)	(2,252.92–2,546.81)	(7,507.89–7,821.28)
Oriental	6,032	5,123.73	1,786.20	6,905.76
		(5,038.31–5,209.15)	(1,663.04–1,905.00)	(6,759.79–7,067.89)
Orinoquía	346	7,934.38	15.98	7,949.72
		(7,568.60–8,300.16)	(11.26–20.73)	(7,600.68–8,334.57)
Pacífica	7,752	6,533.19	4,860.81	11,383.67
		(6,344.92–6,721.45)	(4,558.42–5,152.12)	(11,035.39–11,767.61)
All	46,148	5,142.75	3,229.75	8,366.21
		(5,113.17–5,172.34)	(3,030.17–3,426.80)	(8,169.79–8,573.28)

95% Confidence Intervals in parenthesis, *n* number of cases by region

### Estimation of net costs associated to non-HBP services

The total and mean attributable costs per patient due to the delivery of non-HBP services in the contributory regime are listed in Table 2, and S9 and S10 in the additional file. In summary, the total cost was almost $150 million (95% CI $139.9 to $158.2 million). The mean per patient cost was $3,230 (95% CI $3,030 to $3,427). There were striking differences in these costs between regions, being almost zero in the Orinoquia region and almost $5,000 in the Pacific region. No apparent differences were found between age groups.

### Estimation of total attributable costs

The total cost of delivery of health services in patients with BC in the contributory regime in 2019 was $386.5 million (95% CI $377 to $395.6 million), with a mean per patient cost of $8.366 (95% CI $8,170 to $8,573). The mean per patient costs ranged from $6,906 in the Oriental region to $11,384 in the Pacific region. In parallel with the findings on costs due to the delivery of HBP services, total attributable costs per patient were consistently lower in women of older ages (from $8,429 to $12,646 in the group of 20 to 44 years old to $4,508 to $9,592 in the group of 65 years old or older).

## Discussion

This study estimates the net costs associated to BC in women affiliated with the contributory regime in Colombia in 2019. Total net costs associated to BC and borne by the contributory regime in 2019 ranged from $377 to $396 million, with mean per patient costs that ranged from $6,906 in the Oriental region to $11,384 in the Pacific region. To the best of our knowledge, this is the first published report that estimates the costs of delivering health services in BC using national data from Colombia, and it is also the first to describe differences in these expenditures across the country.

Several studies have evaluated the economic burden that BC imposes on Latin American health systems. Palacios et al., in a systematic review of the literature, summarized existing evidence regarding the health care costs of patients with BC and their relatives in Latin America and the Caribbean [30]. The authors identified 63 studies, 8 of which described the costs of BC in Colombia. Buendia et al., in a cost-effectiveness analysis of trastuzumab for the treatment of early human epidermal growth factor receptor 2-positive (HER2+) BC, estimated the costs of the disease in Colombia in 2012 [13]. Using a Markov model, the authors estimated a lifetime cost of $132,361 per patient treated with trastuzumab and of $75,315 per patient on the control treatment. Likewise, using a Markov model, Chicaíza et al. estimated the cost-effectiveness of trastuzumab in the treatment of epidermal growth factor receptor 2-positive (ErbB2+) metastatic BC [14]. The authors estimated a 5-year cost per patient that ranged from COP $82,017,207 to COP $105,313,611 ($68,144 and $87,500 in 2012 international dollars), depending on the selected treatment strategy. More recently, Perea et al., in an analysis of the cost-effectiveness of immediate versus delayed breast reconstruction, estimated the health care cost of BC with a time horizon of one year [31]. The authors calculated a cost per patient that ranged from $11,060 to $11,165 depending on the type of treatment strategy.

In addition to these model-based estimations, some authors have also used case study methods and an analysis of administrative databases to calculate the costs of BC in Colombia. Gamboa et al., using a case study, estimated the health care costs of BC by clinical stage [11]. The costs per year ranged from $10,531 to $30,826 in 2020 international dollars for stages I and IV, respectively [30]. Similarly, Moreno et al. evaluated the costs of delivering health care services to patients with BC affiliated with an HMO from 2010 to 2014 [15]. The authors estimated a one-year cost per patient ranging from $5,214 to $8,350 depending on the year in the study period. Finally, Franco et al. estimated the costs and resource utilization of health services in patients with advanced HR+/HER2- BC using medical records from 145 patients who initiated first-line treatment from 2012 to 2014 [12]. The mean costs per patient ranged from $1,972 to $2,716 by line of treatment.

In contrast to previous studies, this study finds that the mean per patient costs of health services in BC ranged from seven to almost eleven thousand dollars in the contributory regime during 2019. There are several reasons why these results can provide a more accurate estimate of the costs paid by the health system than previous studies. First, the UPC database provides a rich and reliable source of information on the use of health services by individuals. Given that the data are generated at the point of delivery by providers and are key to all claims made by HMOs to the Ministry of Health, we believe that this is an appropriate incentive to avoid an underreporting of health services. Furthermore, as described in detail by Bolivar et al., the UPC database undergoes an extensive process of data cleaning and cross-checking using, among others, the financial reports that HMOs regularly submit to the Ministry of Health and data from RUAF and BDUA [23]. This external validation process may serve as an additional auditing tool to identify duplications of reported services or individuals within the database.

A second reason why our results may provide more accurate estimates is that this study includes all services delivered to patients with BC, independent of the type of service and the registered diagnosis. This feature is of great importance since many complications due to the disease or treatment might be registered in administrative databases as “unrelated” to the main diagnosis. In contrast, to decrease the risk of excessively allocating services to the main diagnosis, this study uses econometric methods to isolate the effects of the disease on fitted costs [32].

A third reason that further lends support to the accuracy of our results is that the UPC database contains actual data about prices paid by the health system. Given the wide variation in market prices for health services and the extensive bargaining processes that HMOs undertake with individual providers, there may be substantial differences between actual prices paid by the health system and the prices registered in tariff manuals. Using data from UPC allows this study to reduce the reliance of its estimates on unverified assumptions about market prices.

Finally, it is well-known that the analysis of healthcare resources poses some significant challenges. These challenges are based on the fact that, among other things, healthcare data often show substantial positive skewness, heavy tales and are often multimodal (e.g. with a mass at zero for non-users) [33]. This non-normal distribution is frequently associated with the production of non-informative means. Given that, the mean is the statistic of interest to most policy makers [34], this study calculates the means of 2019 BC healthcare expenditures for two previous pre-specified subgroups: the subgroup of individuals diagnosed during 2018 and the subgroup of individuals who died during the first semester of 2020. We acknowledge that this approach does not allow to make inferences about how mean estimates of costs change during the course of the disease, however, we believe that this information may help policy makers to prioritize health programs for patients with recent diagnosis or who are at the proximity of death.

One surprising finding of this study is the important differences between the mean per patient costs identified by region. Several differences in the characteristics of demand for health services in BC across the country may help explain this wide variation in costs. A more advanced stage of the disease at diagnosis, especially in regions with low access or utilization of preventive care, may increase the costs of delivering health services in BC [35, 36]. Likewise, regions with large segments of their populations with earnings below the poverty line and with low levels of schooling may also present less for HBP and particularly non-HBP services [37].

In addition to differences in demand, differences in characteristics of supply may also help explain these large differences in mean per patient costs between regions. The delivery of oncology services in Colombia is highly concentrated in the large urban areas of Bogota, Antioquia and Valle del Cauca [38]. Except for Vaupes, in 2018, all departments from the Orinoquia – Amazonia region did not report any registered providers of oncological services. It is highly likely that markets with a higher concentration of providers may exert considerable pressures to decrease prices and therefore lower the mean per patient costs of delivering health services [39].

Our study has some weaknesses that must be addressed. The first concerns the use of data regarding the clinical stage to estimate disease severity. The study estimates the impact of the severity of BC on the costs of health services in 2019 by exploring differences in cost estimates for patients who died during the first semester of 2020. The impacts of these advanced stages on the costs of health services are shown in Fig. 2. In summary, patients who died during the first semester of 2020 presented an almost three-fold increase in costs compared to patients in the main cohort.

Another weakness of this study is that prices were not actually paid by the health system due to the delivery of non-HBP services. Using prices from tariff manuals ignores the fact that the characteristics of health markets may affect the final costs of services. To incorporate this uncertainty into our estimates, the study used the range of prices identified from tariff manuals to feed 95% confidence intervals for each estimate of costs in non-HBP services.

Another weakness is that unfortunately, we don’t have reliable data regarding the population without data in the UPC database. This population is affiliated with insurance companies whose data do not fulfill the minimum standards for quality and completeness required by the Ministry of Health, and therefore, are not available for research purposes [23]. This population is, most of the times, affiliated with insurance companies with relatively fewer affiliates, at higher financial risk, and whose affiliates live outside the Andes and Caribe regions (see Table S11 in the additional file). Nevertheless, we believe that our approach may provide at least a conservative estimate of the costs of BC in this population. This assumption is based on the fact that, this unfavorable financial conditions is frequently associated with low investments on disease screening and early treatment, and therefore, might result in greater costs due to a higher rate of complications and end-of-life care [40].

Finally, there is no information regarding the histological confirmation of the diagnosis of BC. As mentioned above, to decrease the impact of including patients without the disease (i.e., false-positive cases), the study used the identification algorithm developed by Saldaña et al. [26]. According to these authors, BC identification algorithms have a high PPV compared to identification algorithms for other malignancies. Furthermore, the “specific” algorithm developed by Saldaña et al. for the UPC database provides very close estimates of incidence when compared to official registries of cancer in Colombia [41].

## Conclusions

This study estimates net costs associated to BC that were borne by the contributory regime in 2019. It also describes the distribution of these costs across age groups, regions, and settings of delivery of services. These estimates can help in designing public health interventions aimed at promoting the early detection of BC, making informed decisions on the allocation of health resources, and producing essential data for cost-effectiveness analysis of interventions, among others. This study also provides information that will help improve understanding of the financial effects of BC on health systems from LMICs worldwide. Further research is needed to identify the mechanisms that help explain the remarkable differences in mean per patient costs across the country.

### Electronic supplementary material

Below is the link to the electronic supplementary material.

Supplementary Material 1

## Data Availability

The data that support the findings of this study are available from the Colombian Ministry of Health, but restrictions apply to the availability of these data, which were used under license for the current study, and so are not publicly available. Data are however available from the authors upon reasonable request and with permission of the Colombian Ministry of Health.

## Abbreviations

BC: Breast Cancer
LMICs: Low- and middle-income countries
COI: Cost-of-illness studies
HBP: Health Benefit Package
UPC: Database for the Study of the Sufficiency of the Capitation Unit
ICD-10: International Classification of Diseases, 10th revision
RUAF: Unique Registry of Affiliates
BDUA: Unique Database of Affiliation
MIPRES: My Prescription Database
CI: Confidence Interval
PSM: Propensity Score Matching
CCI: Charlson Comorbidity Index
HER2: Human Epidermal Growth Factor Receptor 2
ErbB2: Epidermal Growth Factor Receptor 2
