# Supplementary material for: Net costs of breast cancer in Colombia: a cost-of-illness study based on administrative claims databases
Source: Cost Eff Resour Alloc. 2024 Jul 2;22:54. doi: 10.1186/s12962-024-00562-z (PMC11218325; doi:10.1186/s12962-024-00562-z)
Supplement: Supplementary file 1 — Supplementary Material 1 [file 12962_2024_562_MOESM1_ESM.docx]

# Supplementary Data

Table S Electronic algorithms used to identify individuals with BC within the UPC database.

| Criteria | Sensitive algorithm | Specific algorithm |
| --- | --- | --- |
|  |  |  |
| At least one of the following ICD-10 codes from 2015 to 2019: C501, C502, C503, C504, C505, C506, C507, C508, C509, C771, C773, D050, D051, C057, D059, D486 | Yes | Yes |
| At least one of the following CUPS codes from 2015 to 2019: 851101, 851102, 851200, 851301, 851302, 851101, 852100, 852200, 852300, 852500, 853300, 853301, 854100, 854200, 854001, 854301, 854400, 854501, 854502, 854600, 854701, 854800, 853302, 854002, 855001, 855002, 855301, 855401, 857100, 857200, 858200, 858300, 857203, 857204, 858401, 858402, 858403, 858405, 858701, 859500, 853102, 857201, 857202, 857102, 853103, 853104, 853201, 853202, 853203, 853204, 853401, 853402, 857101, 402200, 402300, 405100, 402201, 402202, 402210, 402301, 402302, 992503, 549004, 992505, 922444, 922443, 908420, 992504, S22222, 992501, 992509, 992506, 857201, 890287, 890387, 890487, 922441, 922442, 922444, 922445, 922446, 922506, 922444 | Yes | No |
| Persistence of at least one of the ICD-10 codes described above for at least four different months (not necessarily calendar consecutive) from 2015 to 2019 | Yes | Yes |
| Consumption of at least one health service (not necessarily related to BC) from January 1^st^ to December 31^st^, 2019 | Yes | Yes |
| First ICD-10 code (from the ones described above) registered in the UPC database from January 1^st^ to December 31^st^, 2018* | Yes | Yes |
| Death from any cause from January 1^st^ to June 31^st^, 2020** | Yes | Yes |
|  |  |  |

* Apply to the subgroup of individuals with first diagnosis during 2018 only

** Apply to the subgroup of individuals deceased during the first semester of 2020 only

CUPS (Unique Code of Health Procedures): identification code used by the ministry of health to identify each health service delivered in Colombia.

Table S List of non-HBP targeted therapies included in this study.

|  | Number of individuals with at least one prescription * | Mean costs per patient | Total costs |
| --- | --- | --- | --- |
|  |  |  |  |
| Afatinib | 2 | 13,406 | 26,812 |
| Anastrozole | 4,193 | 680 | 2,851,850 |
| Aprepitant | 12 | 653 | 7,832 |
| Bevacizumab | 37 | 82,277 | 3,044,266 |
| Bortezomib | 2 | 7,312 | 14,624 |
| Carboplatin | 283 | 1,098 | 310,733 |
| Cetuximab | 3 | 21,005 | 63,014 |
| Cisplatin | 1 | 77 | 77 |
| Crizotinib | 1 | 38,427 | 38,427 |
| Dabrafenib | 1 | 43,966 | 43,966 |
| Dasatinib | 1 | 18,488 | 18,488 |
| Docetaxel | 2 | 1,318 | 2,636 |
| Doxorubicin | 56 | 4,294 | 240,466 |
| Enzalutamide | 1 | 1,820 | 1,820 |
| Erlotinib | 2 | 3,281 | 6,562 |
| Everolimus | 211 | 70,766 | 14,931,541 |
| Exemestane | 845 | 1,592 | 1,345,053 |
| Fulvestrant | 151 | 7,995 | 1,207,294 |
| Gosereline | 660 | 2,145 | 1,415,615 |
| Ibrutinib | 1 | 45,434 | 45,434 |
| Imatinib | 3 | 10,920 | 32,761 |
| Ixabepilone | 141 | 30,067 | 4,239,458 |
| Lapatinib | 140 | 30,096 | 4,213,423 |
| Lenalidomide | 4 | 12,020 | 48,079 |
| Letrozole | 4,585 | 530 | 2,428,120 |
| Leuprolide acetate | 49 | 1,328 | 65,086 |
| Megestrol acetate | 11 | 1,037 | 11,412 |
| Methylprednisolone | 1 | 69 | 69 |
| Methotrexate | 1 | 272 | 272 |
| Nivolumab | 3 | 116,340 | 349,020 |
| Olaparib | 16 | 89,535 | 1,432,565 |
| Osimertinib | 1 | 54,010 | 54,010 |
| Palbociclib | 846 | 66,450 | 56,216,782 |
| Panitumumab | 1 | 7,085 | 7,085 |
| Pazopanib | 2 | 11,659 | 23,318 |
| Pembrolizumab | 16 | 57,774 | 924,386 |
| Pemetrexed | 4 | 7,182 | 28,726 |
| Pertuzumab | 834 | 3,049 | 2,543,085 |
| Raloxifen | 4 | 388 | 1,553 |
| Ribociclib | 276 | 60,031 | 16,568,431 |
| Rituximab | 1 | 4,304 | 4,304 |
| Sorafenib | 5 | 20,701 | 103,507 |
| Sunitinib | 1 | 50,467 | 50,467 |
| Tamoxifen | 1 | 669 | 669 |
| Temozolomide | 1 | 782 | 782 |
| Trabectedine | 1 | 3,570 | 3,570 |
| Trametinib dimethylsulfoxide | 1 | 31,325 | 31,325 |
| Trastuzumab | 45 | 7,188 | 323,451 |
| Trastuzumab emtansine | 479 | 70,420 | 33,731,010 |
| Vinorelbine | 1 | 474 | 474 |
|  |  |  |  |

* Includes all available pharmaceutical presentations

Table S Sensitivity analysis: Marginal effects estimated using the full cohort (with employment data only) and using the subgroup with known income data

|  | Eployment data only | Additional income data |
| --- | --- | --- |
|  | n=7,845,081 | n=6,213,174 |
|  |  |  |
| Main cohort |  |  |
| All costs | 5,142.75 | 5,757.79 |
|  | (5,113.17 - 5,172.34) | (5,248.05 - 6,267.53) |
| Inpatient | 974.04 | 1,073.89 |
|  | (955.18 - 992.89) | (889.87 - 1,257.90) |
| Outpatient | 4,177.76 | 4,651.11 |
|  | (4,160.28 - 4,195.25) | (4,268.24 - 5,033.99) |
| First diagnosis during 2018 | |  |
| All costs | 13,389.85 | 15,001.21 |
|  | (13,312.28 - 13,467.41) | (13,965.72 - 16,036.70) |
| Inpatient | 2,584.99 | 2,704.59 |
|  | (2,535.62 - 2,634.37) | (2,133.66 - 3,275.52) |
| Outpatient | 10,720.87 | 12,181.71 |
|  | (10,674.18 - 10,767.57) | (11,592.93 - 12,770.49) |
| Deceased during the first semester of 2020 | |  |
| All costs | 13,505.96 | 16,539.92 |
|  | (13,314.25 - 13,697.66) | (15,030.42 - 18,049.42) |
| Inpatient | 4,201.87 | 5,177.78 |
|  | (4,080.52 - 4,323.22) | (3,667.68 - 6,687.87) |
| Outpatient | 8,794.46 | 10,859.98 |
|  | (8,678.44 - 8,910.48) | (9,941.33 - 11,778.63) |
|  |  |  |

95% Confidence Interval in parenthesis

Table S Characteristics of the individuals before and after matching

|  | Before matching | | After marching | | Remaining bias (%) | Bias reduction (%) |
| --- | --- | --- | --- | --- | --- | --- |
| Variable | Treated | Control | Treated | Control |  |  |
|  |  |  |  |  |  |  |
| Main cohort | | | | | | |
| Age | 58.62 | 46.15 | 58.61 | 58.70 | -0.6 | 99.2 |
| Bogotá | 0.29 | 0.32 | 0.30 | 0.30 | -0.7 | 85.7 |
| Central | 0.31 | 0.27 | 0.31 | 0.31 | -1 | 88 |
| Oriental | 0.13 | 0.14 | 0.13 | 0.12 | 1.2 | 75.5 |
| Orinoquía | 0.01 | 0.14 | 0.01 | 0.01 | 0.2 | 99.7 |
| Pacífica | 0.16 | 0.01 | 0.16 | 0.16 | -0.1 | 99.8 |
| CCI I | 0.24 | 0.07 | 0.24 | 0.25 | -3 | 93.9 |
| CCI II | 0.11 | 0.02 | 0.11 | 0.10 | 3.3 | 91.3 |
| CCI III | 0.07 | 0.01 | 0.07 | 0.06 | 5 | 84.3 |
| Employed* | 0.66 | 0.64 | 0.66 | 0.67 | -3 | -12.6 |
|  |  |  |  |  |  |  |
| First diagnosis during 2018 | | | | | | |
| Age | 57.53 | 46.20 | 57.53 | 57.57 | -0.2 | 99.7 |
| Bogotá | 0.27 | 0.32 | 0.27 | 0.27 | -0.2 | 98.2 |
| Central | 0.31 | 0.27 | 0.31 | 0.31 | -0.1 | 98.4 |
| Oriental | 0.14 | 0.14 | 0.14 | 0.14 | 0.2 | 78.3 |
| Orinoquía | 0.01 | 0.14 | 0.01 | 0.01 | -0.1 | 99.8 |
| Pacífica | 0.16 | 0.01 | 0.16 | 0.16 | 0.1 | 99.9 |
| CCI I | 0.22 | 0.07 | 0.22 | 0.22 | -0.6 | 98.6 |
| CCI II | 0.10 | 0.02 | 0.10 | 0.10 | 1 | 97.1 |
| CCI III | 0.06 | 0.01 | 0.06 | 0.06 | 1.2 | 95.7 |
| Employed* | 0.64 | 0.64 | 0.64 | 0.64 | -0.6 | 22.1 |
|  |  |  |  |  |  |  |
| Deceased during the first semester of 2020 | | | | | | |
| Age | 62.17 | 46.21 | 62.17 | 61.93 | 1.4 | 98.5 |
| Bogotá | 0.28 | 0.32 | 0.28 | 0.28 | -0.7 | 92 |
| Central | 0.28 | 0.27 | 0.28 | 0.28 | 0.3 | 84.2 |
| Oriental | 0.14 | 0.14 | 0.14 | 0.14 | 0.4 | 59.3 |
| Orinoquía | 0.00 | 0.14 | 0.00 | 0.00 | 0 | 100 |
| Pacífica | 0.17 | 0.01 | 0.17 | 0.17 | -0.5 | 99.1 |
| CCI I | 0.30 | 0.07 | 0.30 | 0.30 | -0.4 | 99.3 |
| CCI II | 0.17 | 0.02 | 0.17 | 0.17 | 0.5 | 99 |
| CCI III | 0.11 | 0.01 | 0.11 | 0.11 | 0.6 | 98.5 |
| Employed* | 0.62 | 0.64 | 0.62 | 0.62 | -0.3 | 94.8 |
|  |  |  |  |  |  |  |

Table S Prevalence of Breast Cancer per 1,000 women affiliated with the contributory regime by region and age group, conditional to the consumption of at least one health service during 2019.

|  | Main Cohort | | First diagnosis during 2018 | | Deceased during the first semester of 2020 | |
| --- | --- | --- | --- | --- | --- | --- |
| Age group  (in years) | Sensitive algorithm* | Specific algorithm* | Sensitive algorithm* | Specific algorithm* | Sensitive algorithm* | Specific algorithm* |
|  |  |  |  |  |  |  |
| Atlantica |  |  |  |  |  |  |
| 20 a 44 | 2.649 | 1.323 | 0.228 | 0.212 | 0.024 | 0.024 |
| 45 a 64 | 12.465 | 8.097 | 1.270 | 1.108 | 0.185 | 0.181 |
| 65 or more | 21.545 | 11.819 | 1.956 | 1.473 | 0.514 | 0.383 |
| Bogota |  |  |  |  |  |  |
| 20 a 44 | 2.359 | 1.685 | 0.219 | 0.197 | 0.021 | 0.020 |
| 45 a 64 | 11.994 | 8.353 | 1.174 | 0.998 | 0.183 | 0.148 |
| 65 or more | 20.170 | 11.589 | 1.516 | 1.168 | 0.495 | 0.320 |
| Central |  |  |  |  |  |  |
| 20 a 44 | 1.690 | 1.197 | 0.228 | 0.210 | 0.022 | 0.022 |
| 45 a 64 | 12.846 | 9.266 | 1.357 | 1.241 | 0.149 | 0.133 |
| 65 or more | 22.164 | 14.216 | 1.805 | 1.520 | 0.441 | 0.337 |
| Oriental |  |  |  |  |  |  |
| 20 a 44 | 2.135 | 1.075 | 0.190 | 0.172 | 0.032 | 0.028 |
| 45 a 64 | 11.244 | 7.475 | 1.201 | 1.080 | 0.154 | 0.139 |
| 65 or more | 19.562 | 11.172 | 1.411 | 1.244 | 0.562 | 0.314 |
| Orinoquia - Amazonia |  |  |  |  |  |  |
| 20 a 44 | 5.231 | 1.213 | 0.214 | 0.190 | 0.048 | 0.048 |
| 45 a 64 | 14.009 | 6.800 | 1.176 | 1.074 | 0.051 | 0.051 |
| 65 or more | 16.732 | 7.087 | 0.591 | 0.591 | 0.197 | 0.197 |
| Pacifica |  |  |  |  |  |  |
| 20 a 44 | 2.570 | 1.657 | 0.235 | 0.212 | 0.029 | 0.027 |
| 45 a 64 | 12.492 | 9.206 | 1.327 | 1.183 | 0.159 | 0.153 |
| 65 or more | 21.603 | 14.042 | 2.043 | 1.658 | 0.600 | 0.453 |
|  |  |  |  |  |  |  |

* As described in Saldaña et al [^1^](#_ENREF_1).

Table S Estimated cases of Breast Cancer in the contributory regime by region and age group, conditional to the consumption of at least one health service during 2019

|  |  | Main Cohort | | First diagnosis during 2018 | | Deceased during the first semester of 2020 | |
| --- | --- | --- | --- | --- | --- | --- | --- |
| Age group  (in years) | Affiliates | Sensitive algorithm* | Specific algorithm* | Sensitive algorithm* | Specific algorithm* | Sensitive algorithm* | Specific algorithm* |
|  |  |  |  |  |  |  |  |
| Atlantica |  |  |  |  |  |  |  |
| 20 a 44 | 618,075 | 1,637 | 818 | 141 | 131 | 15 | 15 |
| 45 a 64 | 320,093 | 3,990 | 2,592 | 407 | 355 | 59 | 58 |
| 65 or more | 150,609 | 3,245 | 1,780 | 295 | 222 | 77 | 58 |
| Bogota |  |  |  |  |  |  |  |
| 20 a 44 | 1,396,138 | 3,293 | 2,353 | 306 | 275 | 29 | 28 |
| 45 a 64 | 775,276 | 9,299 | 6,476 | 910 | 774 | 142 | 114 |
| 65 or more | 350,166 | 7,063 | 4,058 | 531 | 409 | 173 | 112 |
| Central |  |  |  |  |  |  |  |
| 20 a 44 | 1,269,071 | 2,144 | 1,519 | 289 | 267 | 28 | 28 |
| 45 a 64 | 766,429 | 9,845 | 7,102 | 1,040 | 951 | 115 | 102 |
| 65 or more | 374,368 | 8,298 | 5,322 | 676 | 569 | 165 | 126 |
| Oriental |  |  |  |  |  |  |  |
| 20 a 44 | 790,150 | 1,687 | 849 | 150 | 136 | 26 | 22 |
| 45 a 64 | 420,275 | 4,725 | 3,142 | 505 | 454 | 65 | 58 |
| 65 or more | 182,698 | 3,574 | 2,041 | 258 | 227 | 103 | 57 |
| Orinoquia - Amazonia |  |  |  |  |  |  |  |
| 20 a 44 | 74,569 | 390 | 90 | 16 | 14 | 4 | 4 |
| 45 a 64 | 30,068 | 421 | 204 | 35 | 32 | 2 | 2 |
| 65 or more | 7,194 | 120 | 51 | 4 | 4 | 1 | 1 |
| Pacifica |  |  |  |  |  |  |  |
| 20 a 44 | 642,850 | 1,652 | 1,065 | 151 | 136 | 18 | 17 |
| 45 a 64 | 399,878 | 4,995 | 3,681 | 531 | 473 | 64 | 61 |
| 65 or more | 214,056 | 4,624 | 3,006 | 437 | 355 | 128 | 97 |
|  |  |  |  |  |  |  |  |
| Total | 8,781,963 | 71,004 | 46,148 | 6,680 | 5,784 | 1,213 | 960 |
|  |  |  |  |  |  |  |  |

* As described in Saldaña et al [^1^](#_ENREF_1).

Table S Total costs of delivering HBP health services per category of service

|  | Quantities per patient | | Costs per patient | | Total borne by the health system |
| --- | --- | --- | --- | --- | --- |
|  | Mean | SD | Mean | SD |  |
| n = 46,149 |  |  |  |  |  |
| Outpatient |  |  |  |  |  |
| Consultations | 13 | 10 | 413 | 660 | 19,042,553 |
| Diagnostic imaging | 4.5 | 3.84 | 406 | 663 | 18,740,307 |
| Laboratory | 22.94 | 24.21 | 448 | 1,314 | 20,677,682 |
| Other* | 38.13 | 35.41 | 4,414 | 9,471 | 203,696,446 |
| Inpatient |  |  |  |  |  |
| General ward | 0.58 | 1.63 | 369 | 2,318 | 17,012,778 |
| Intensive care | 0.04 | 0.35 | 118 | 1,771 | 5,433,290 |
| Emergency | 0.74 | 1.49 | 122 | 843 | 5,648,883 |
| Total |  |  | 7,654 | 12,889 | 290,251,938 |
|  |  |  |  |  |  |

* Includes other outpatient services such as: ambulatory surgical procedures, oncology infusion services, ambulatory medications, among others.

Table S Adjusted costs and marginal effects of exposed and unexposed individuals

|  | Adjusted costs | | Marginal effect |
| --- | --- | --- | --- |
|  | Exposed | Unexposed | (95% Confidence Interval) |
|  |  |  |  |
| Main cohort |  |  |  |
| All costs | 5,761 | 618 | 5,142.75 |
|  |  |  | (5,113.17 - 5,172.34) |
| Inpatient | 1,072 | 98 | 974.04 |
|  |  |  | (955.18 - 992.89) |
| Outpatient | 4,642 | 465 | 4,177.76 |
|  |  |  | (4,160.28 - 4,195.25) |
| First diagnosis during 2018 | |  |  |
| All costs | 13,724 | 334 | 13,389.85 |
|  |  |  | (13,312.28 - 13,467.41) |
| Inpatient | 2,630 | 45 | 2,584.99 |
|  |  |  | (2,535.62 - 2,634.37) |
| Outpatient | 10,950 | 230 | 10,720.87 |
|  |  |  | (10,674.18 - 10,767.57) |
| Deceased during the first semester of 2020 | | |  |
| All costs | 13,731 | 225 | 13,505.96 |
|  |  |  | (13,314.25 - 13,697.66) |
| Inpatient | 4,228 | 26 | 4,201.87 |
|  |  |  | (4,080.52 - 4,323.22) |
| Outpatient | 8,933 | 139 | 8,794.46 |
|  |  |  | (8,678.44 - 8,910.48) |
|  |  |  |  |

Table S Total costs of delivering health services in patients with Breast Cancer affiliated with the Contributory Regime in 2019

| Age group  (in years) | n | Services included in the Health Benefit Package | Services not included in the Health Benefit Package | Total |
| --- | --- | --- | --- | --- |
|  |  |  |  |  |
| Atlántico |  |  |  |  |
| 20 a 44 | 818 | 6,000,204.44 | 2,371,246.00 | 8,356,821.79 |
|  |  | (5,289,015.62 - 6,798,050.85) | (2,318,331.32 - 2,422,222.51) | (8,240,158.33 - 8,470,624.84) |
| 45 a 64 | 2,592 | 14,572,414.01 | 10,214,926.87 | 24,875,163.47 |
|  |  | (12,563,152.45 - 16,912,378.12) | (8,980,474.86 - 11,425,720.05) | (23,577,869.39 - 26,213,217.52) |
| 65 or more | 1,780 | 5,067,529.43 | 4,409,410.01 | 9,524,706.61 |
|  |  | (3,772,155.76 - 6,433,795.28) | (3,744,864.14 - 5,077,472.72) | (8,753,431.70 - 10,336,325.14) |
| Bogotá |  |  |  |  |
| 20 a 44 | 2,353 | 13,354,234.10 | 6,488,496.30 | 19,841,009.47 |
|  |  | (13,161,136.79 - 13,555,448.59) | (6,416,853.68 - 6,558,360.70) | (19,631,243.10 - 20,040,231.13) |
| 45 a 64 | 6,476 | 34,218,944.24 | 25,488,512.54 | 59,708,980.44 |
|  |  | (33,745,529.71 - 34,723,013.90) | (24,188,966.23 - 26,763,734.51) | (58,299,722.60 - 61,171,171.21) |
| 65 or more | 4,058 | 13,887,247.66 | 18,299,382.16 | 32,159,796.30 |
|  |  | (13,316,062.74 - 14,405,257.10) | (17,381,562.02 - 19,233,812.03) | (31,137,229.97 - 33,313,350.09) |
| Central |  |  |  |  |
| 20 a 44 | 1,519 | 13,290,444.19 | 4,307,245.19 | 17,567,207.29 |
|  |  | (12,002,952.28 - 14,712,153.09) | (3,964,203.89 - 4,640,595.94) | (17,184,710.67 - 17,941,624.79) |
| 45 a 64 | 7,102 | 39,672,192.29 | 17,052,872.00 | 57,067,027.34 |
|  |  | (32,647,303.91 - 48,103,432.76) | (16,343,790.82 - 17,766,356.62) | (56,168,634.42 - 57,984,838.0) |
| 65 or more | 5,322 | 18,630,314.60 | 12,064,775.32 | 30,743,125.96 |
|  |  | (13,776,954.45 - 23,768,661.02) | (11,025,518.40 - 13,143,570.00) | (29,568,019.93 - 32,089,277.69) |
|  |  |  |  |  |
| Oriental |  |  |  |  |
| 20 a 44 | 849 | 6,736,600.73 | 1,638,342.96 | 8,367,010.23 |
|  |  | (4,890,926.57 - 9,075,867.61) | (1,544,324.56 - 1,729,170.55) | (8,229,804.08 - 8,510,235.01) |
| 45 a 64 | 3,142 | 17,715,483.46 | 5,583,921.78 | 23,404,622.48 |
|  |  | (15,253,694.84 - 20,584,802.79) | (5,117,165.12 - 6,044,363.06) | (22,739,117.47 - 24,044,445.12) |
| 65 or more | 2,041 | 5,542,619.73 | 3,537,273.92 | 9,050,517.42 |
|  |  | (3,576,743.52 - 7,789,530.13) | (3,343,610.24 - 3,736,568.49) | (8,471,665.08 - 9,668,349.88) |
| Orinoquía - Amazonía |  |  |  |  |
| 20 a 44 | 1,065 | 903,442.62 | 0.00 | 910,914.30 |
|  |  | (510,066.92 - 1,481,285.13) | - | (860,825.64 - 963,016.33) |
| 45 a 64 | 3,681 | 1,501,586.83 | 5,367.56 | 1,553,502.06 |
|  |  | (950,472.15 - 2,279,320.97) | (3,742.82 - 6,988.70) | (1,452,235.42 - 1,652,198.30) |
| 65 or more | 3,006 | 226,884.69 | 164.95 | 228,298.15 |
|  |  | (120,545.04 - 361,793.92) | (49.49 - 283.97) | (166,536.98 - 301,351.31) |
| Pacífico |  |  |  |  |
| 20 a 44 | 90 | 9,831,108.40 | 3,653,438.00 | 13,512,179.35 |
|  |  | (8,909,287.46 - 10,846,739.62) | (3,478,875.37 - 3,821,210.78) | (13,130,632.19 - 13,898,664.30) |
| 45 a 64 | 204 | 27,297,576.75 | 18,636,483.78 | 46,126,995.98 |
|  |  | (22,736,336.84 - 32,729,640.21) | (17,548,441.21 - 19,708,305.41) | (44,327,589.60 - 47,871,863.52) |
| 65 or more | 51 | 13,336,877.13 | 15,337,374.30 | 27,060,007.74 |
|  |  | (8,369,761.02 - 19,101,693.08) | (14,282,340.12 - 16,433,868.26) | (24,432,944.24 - 29,953,996.43) |
|  |  |  |  |  |
| All | 46,148 | 237,338,066.46 | 149,072,153.71 | 386,531,458.16 |
|  |  | (236,000,630.06 - 238,739,327.81) | (139,885,147.51 - 158,238,956.82) | (377,046,902.22 - 395,965,989.86) |
|  |  |  |  |  |

95% Confidence Intervals in parenthesis

Table S Mean attributable costs per patient in the Contributory Regime during 2019 by region and age group.

| Age group  (in years) | n | Services included in the Health Benefit Package | Services not included in the Health Benefit Package | Total |
| --- | --- | --- | --- | --- |
|  |  |  |  |  |
| Atlántico |  |  |  |  |
| 20 a 44 | 818 | 7,318.93 | 2,898.68 | 10,218.75 |
|  |  | (7,198.31 - 7,439.55) | (2,834.56 - 2,960.41) | (10,079.57 - 10,359.73) |
| 45 a 64 | 2,592 | 5,658.69 | 3,936.34 | 9,579.86 |
|  |  | (5,533.90 - 5,783.49) | (3,464.93 - 4,405.41) | (9,102.19 - 10,111.45) |
| 65 or more | 1,780 | 2,873.80 | 2,479.30 | 5,352.87 |
|  |  | (2,680.05 - 3,067.56) | (2,103.52 - 2,851.62) | (4,912.15 - 5,781.62) |
| Bogotá |  |  |  |  |
| 20 a 44 | 2,353 | 5,674.33 | 2,758.09 | 8,432.82 |
|  |  | (5,593.19 - 5,755.47) | (2,728.46 - 2,787.32) | (8,344.47 - 8,520.22) |
| 45 a 64 | 6,476 | 5,286.04 | 3,935.15 | 9,220.18 |
|  |  | (5,209.78 - 5,362.31) | (3,735.44 - 4,132.69) | (9,002.44 - 9,444.95) |
| 65 or more | 4,058 | 3,422.39 | 4,510.29 | 7,930.81 |
|  |  | (3,289.66 - 3,555.12) | (4,280.62 - 4,735.35) | (7,665.40 - 8,196.74) |
| Central |  |  |  |  |
| 20 a 44 | 1,519 | 8,731.64 | 2,835.69 | 11,571.30 |
|  |  | (8,634.78 - 8,828.51) | (2,609.93 - 3,053.12) | (11,327.17 - 11,818.23) |
| 45 a 64 | 7,102 | 5,634.18 | 2,401.45 | 8,035.29 |
|  |  | (5,557.43 - 5,710.92) | (2,300.92 - 2,501.31) | (7,912.89 - 8,167.08) |
| 65 or more | 5,322 | 3,517.50 | 2,269.42 | 5,784.22 |
|  |  | (3,399.98 - 3,635.01) | (2,069.21 - 2,469.38) | (5,544.76 - 6,019.25) |
| Oriental |  |  |  |  |
| 20 a 44 | 849 | 7,927.02 | 1,929.18 | 9,856.95 |
|  |  | (7,807.03 - 8,047.01) | (1,818.14 - 2,035.64) | (9,692.97 - 10,028.46) |
| 45 a 64 | 3,142 | 5,671.00 | 1,775.71 | 7,446.05 |
|  |  | (5,546.05 - 5,795.95) | (1,627.76 - 1,923.28) | (7,243.51 - 7,659.01) |
| 65 or more | 2,041 | 2,705.82 | 1,733.56 | 4,433.58 |
|  |  | (2,437.78 - 2,973.85) | (1,637.28 - 1,829.24) | (4,139.41 - 4,725.69) |
| Pacífico |  |  |  |  |
| 20 a 44 | 1,065 | 10,069.62 | 3,430.05 | 12,693.91 |
|  |  | (9,505.53 - 10,633.70) | (3,267.00 - 3,587.51) | (12,334.33 - 13,066.73) |
| 45 a 64 | 3,681 | 7,564.26 | 5,059.65 | 12,525.84 |
|  |  | (7,077.16 - 8,051.35) | (4,767.20 - 5,351.65) | (12,082.79 - 13,012.60) |
| 65 or more | 3,006 | 4,520.50 | 5,106.21 | 9,002.43 |
|  |  | (3,218.36 - 5,822.64) | (4,746.36 - 5,466.45) | (8,091.60 - 9,938.79) |
| Orinoquía-Amazonía |  |  |  |  |
| 20 a 44 | 90 | 9,262.42 | 0.00 | 10,073.66 |
|  |  | (8,952.44 - 9,572.39) | ( - ) | (9,519.74 - 10,649.85) |
| 45 a 64 | 204 | 7,465.09 | 26.18 | 7,598.87 |
|  |  | (7,119.90 - 7,810.28) | (18.35 - 34.27) | (7,101.92 - 8,079.65) |
| 65 or more | 51 | 3,919.29 | 3.26 | 4,477.59 |
|  |  | (3,123.72 - 4,714.87) | (0.99 - 5.57) | (3,266.88 - 5,913.52) |
|  |  |  |  |  |

95% Confidence Intervals in parenthesis

Table S Characteristics of the population with and without data included in the UPC database.

| Variable | Population with data  available in the UPC database | Population without data available in the UPC database | Mean difference  (in percentage points) |
| --- | --- | --- | --- |
|  | N (%) | N (%) |  |
|  |  |  |  |
| Age [mean (SD)] | 33.84 (19.661) | 35.78 (20.89) | -1.23 |
| Female | 8,481,471 (0.519) | 2,357,042 (0.489) | 3 |
| Region of residence |  |  |  |
| Andina | 11,462,282 (70.1) | 3,118,139 (64.7) | 5 |
| Caribe | 2,318,632 (14.1) | 561,801 (11.6) | 3 |
| Pacífico | 2,207,367 (13.5) | 739,101 (15.3) | -2 |
| Orinoquía | 266,116 (1.6) | 329,253 (6.8) | -5 |
| Amazonía | 75,553 (0.4) | 70,951 (1.4) | -1 |
| Total | 16,329,954 (77.2) | 4,819,486 (22.787) |  |
|  |  |  |  |

References

1. Saldaña Espinel LE, Patiño Benavidez AF, Rozo Agudelo N, et al. Estimating breast, stomach, and colorectal cancer incidence in Colombia through administrative database algorithms: A systematic review of literature and real-world data study. Journal of Clinical Oncology 2021;39:e18810-e.
